# Aetiology and risk factors of musculoskeletal disorders in physically active conscripts: a follow-up study in the Finnish Defence Forces

**DOI:** 10.1186/1471-2474-11-146

**Published:** 2010-07-05

**Authors:** Henri Taanila, Jaana Suni, Harri Pihlajamäki, Ville M Mattila, Olli Ohrankämmen, Petteri Vuorinen, Jari Parkkari

**Affiliations:** 1 Tampere Research Centre of Sports Medicine, the UKK Institute, PO Box 30 33501 Tampere, Finland; 2 Research Department, Centre for Military Medicine, Lahti and Helsinki, Finland; 3 General Headquarters of Finnish Defence Forces, Helsinki, Finland; 4 Staff Department, Pori Brigade, Säkylä, Finland; 5 Research Unit of Pirkanmaa Hospital District and Department of Trauma, Musculoskeletal Surgery and Rehabilitation, Tampere University Hospital, Tampere, Finland

## Abstract

**Background:**

Musculoskeletal disorders (MSDs) are the main reason for morbidity during military training. MSDs commonly result in functional impairment leading to premature discharge from military service and disabilities requiring long-term rehabilitation. The purpose of the study was to examine associations between various risk factors and MSDs with special attention to the physical fitness of the conscripts.

**Methods:**

Two successive cohorts of 18 to 28-year-old male conscripts (*N *= 944, median age 19) were followed for six months. MSDs, including overuse and acute injuries, treated at the garrison clinic were identified and analysed. Associations between MSDs and risk factors were examined by multivariate Cox's proportional hazard models.

**Results:**

During the six-month follow-up of two successive cohorts there were 1629 MSDs and 2879 health clinic visits due to MSDs in 944 persons. The event-based incidence rate for MSD was 10.5 (95% confidence interval (CI): 10.0-11.1) per 1000 person-days. Most MSDs were in the lower extremities (65%) followed by the back (18%). The strongest baseline factors associated with MSDs were poor result in the combined outcome of a 12-minute running test and back lift test (hazard ratio (HR) 2.9; 95% CI: 1.9-4.6), high waist circumference (HR 1.7; 95% CI: 1.3-2.2), high body mass index (HR 1.8; 95% CI: 1.3-2.4), poor result in a 12-minute running test (HR 1.6; 95% CI: 1.2-2.2), earlier musculoskeletal symptoms (HR 1.7; 95% CI: 1.3-2.1) and poor school success (educational level and grades combined; HR 2.0; 95% CI: 1.3-3.0). In addition, risk factors of long-term MSDs (≥10 service days lost due to one or several MSDs) were analysed: poor result in a 12-minute running test, earlier musculoskeletal symptoms, high waist circumference, high body mass index, not belonging to a sports club and poor result in the combined outcome of the 12-minute running test and standing long jump test were strongly associated with long-term MSDs.

**Conclusions:**

The majority of the observed risk factors are modifiable and favourable for future interventions. An appropriate intervention based on the present study would improve both aerobic and muscular fitness prior to conscript training. Attention to appropriate waist circumference and body mass index would strengthen the intervention. Effective results from well-planned randomised controlled studies are needed before initiating large-scale prevention programmes in a military environment.

## Background

Musculoskeletal injuries and disorders are the main reason for morbidity and temporary disability in military populations [1,2]. Health clinic visit rates are approximately equal for injuries and illnesses in the military environment, but the morbidity associated with injuries is over five times greater than that associated with illness [1,3,4]. A recently published hospital discharge register-based study emphasises that injuries are a major cause of morbidity in the Finnish Defence Forces [2]. During the 10-year study period, the incidence of traumatic injury hospitalisation was 94 per 1000 conscripts per year. Moreover, musculoskeletal disorders (MSDs) are the second highest reason for premature discharge from military service in the Finnish Defence Forces, and their number increased clearly at the turn of the millennium [5]. Military service in Finland is compulsory for all male citizens over 18 years of age, the duration varying from six to twelve months. Given that 80% of young men in Finland complete their service period, the high number of MSDs affects public health [2].

Previous epidemiological studies report that several risk factors are associated with injuries during military training. These include, amongst others: female gender [6-9], Caucasian race [10-12], biomechanical factors such as foot structure and flexibility [1,7,11], previous history of injury, high running mileage, high amount of weekly exercise [3,4,13-17], tobacco use [7,11,18,19] and low levels of physical fitness and activity [3,7,10,14,20-24]. The evidence is contradictory, however, with respect to some factors, including age, foot structure, muscular strength and body composition [3,6,7,11,12,21,24,25]. Older age is associated with a higher risk for injuries in most studies [1,8,11,21,24,26], but conflicting results are also reported [3,25,27]. Despite the large number of injuries, there is a lack of epidemiological data concerning the causes and risk factors for musculoskeletal injuries or disorders during conscription military service [9]. In addition, the study populations have been rather small with a short follow-up time [21,23,26,28]. Professional soldiers in the United States (US) have been the major target of injury research in the army environment [1,3,4,10,11], but these results are not directly comparable with those of a conscription army. The number of conscripts, their quality and motivation, as well as practices and training schedules differ substantially in the professional army.

The purpose of the present prospective six-month follow-up study of two successive arrivals was to examine associations between MSDs and various intrinsic risk factors with special attention to the physical fitness of the conscripts. The general hypothesis is that low levels of physical fitness and detrimental health behaviour factors prior to conscription are associated with MSDs during military training.

## Methods

### Subjects

The subjects of this study comprised male conscripts (*N *= 944) from six companies of one brigade (Pori Brigade, Säkylä) in the Finnish Defence Forces. The six companies enrolled into the study were: the anti-tank company, the signal company, the mortar company, the engineer company, the infantry company and the logistic company. In addition, 16 conscripts in the sample were moved to different brigades. During the study period, two arrivals of conscripts started service in the brigade: 359 in July 2006 and 619 in January 2007. The Pori Brigade is a typical Finnish garrison and the chosen companies form a representative sample of conscripts. The baseline characteristics of the companies are presented in Table 1.

**Table 1:** Baseline characteristics of 944 male conscripts by company.

Variable	Anti-Tank company	Signal company	Mortar company	Engineer company	Infantry company	Logistic company	**Other companies**^**1**^	Missing	**P-value**^**2**^
Number of conscripts	249	234	69	215	100	61	16	0 (0%)	-
Age, median, years	19	19	19	19	19	19	19	0 (0%)	0.839^3^
Body mass index, median, kg/m^2^	23.5	22.2	23.5	23.5	22.1	22.8	23.1	75 (8%)	0.025^3^
Waist circumference, median, cm	87.0	85.0	89.0	86.4	84.0	85.0	85.3	51 (5%)	0.015^3^
12-minute run test result, median, m	2320	2395	2530	2408	2388	2250	2535	19 (2%)	<0.001^3^
Muscle fitness index (MFI)^5^, median, points	7	7	9	7	6	6	9	10 (1%)	0.005^3^
Conscript's physical fitness index (CPFI)^6^,median, points	15.25	15.29	16.75	15.58	15.00	14.50	18.18	21 (2%)	<0.001^3^
	Yes	Yes	Yes	Yes	Yes	Yes	Yes		
High level of education^7^,%	48%	35%	46%	39%	36%	48%	56%	10 (1%)	0.037^4^
High level of previous physical activity^8^,%	31%	28%	43%	39%	17%	18%	50%	10 (1%)	<0.001^4^
Good self-assessed health^9^,%	56%	54%	66%	53%	41%	41%	75%	10 (1%)	0.005^4^
Chronic impairment or disability,%	17%	11%	16%	17%	12%	17%	13%	15 (2%)	0.523^4^
Clear musculoskeletal symptoms^10^,%	27%	32%	21%	28%	37%	31%	19%	11 (1%)	0.283^4^

^1 ^Conscript was moved to a different brigade.

^2 ^P-value for difference between the companies.

^3 ^P-value was examined by using a Kruskall-Wallis test for median difference.

^4 ^P-value was examined by using χ^2 ^statistics for difference.

^5 ^MFI is the sum of individual muscle fitness test results comprising push-up, sit-up, pull-up, standing long jump and back lift tests (Excellent = 13-15 points, Good = 9-12 points, Fair good = 5-8 points, Poor = 0-4 points).

^6 ^CPFI = (12 minute running test result (metres) + 100 × MFI)/200, (Excellent [CPFI ≥ 21.00], Good [17.00 ≤ CPFI < 21.00], Fair good [13.00 ≤ CPFI < 17.00], Poor [CPFI < 13.00]).

^7 ^Graduated or studies in higher education institution.

^8 ^Sweating exercise at least three times per week during the last month before military entry.

^9 ^Compared to age-mates.

^10 ^Symptoms lasting more than seven days in at least one anatomical region during the last month before entering the military.

**Figure 1:**
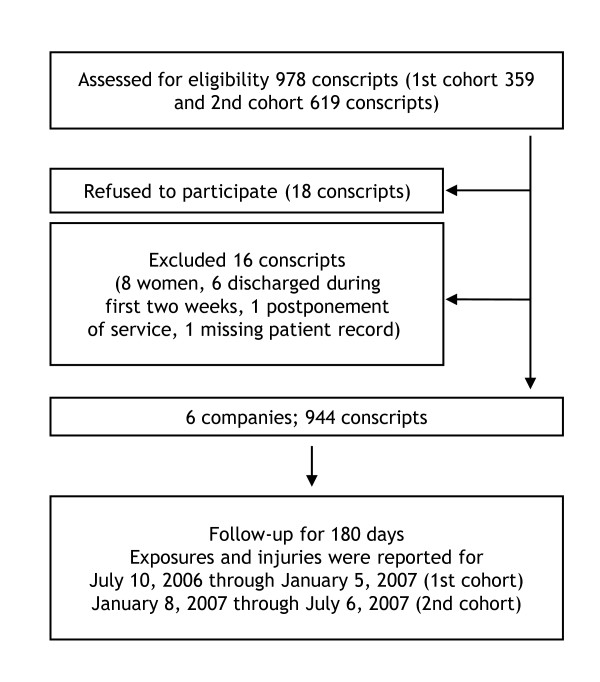
**Flow of conscripts through study**.

The health status of the conscripts was checked during the first two weeks of service by routine medical screenings performed by a physician. Five participants were discharged temporarily (for at least 12 months) and one was discharged permanently from the military service for medical reasons. Because there were only eight women in the study (<1%), they were excluded from the data. In addition, one conscript applied for postponement of the service during the first two weeks and one patient record was missing. Eighteen (<2%) of 962 conscripts refused to participate in the study (Figure 1). All of the remaining conscripts (*N *= 944) agreed to participate and provided their informed consent before the initiation of the study. The group of participants was nearly the same as in a previous descriptive study by the same authors [29]. The age of the conscripts varied from 18 to 28 years (median 19). All subjects were followed for six months beginning on the first day of service. Conscripts who were discharged from the military after the two-week run-in period were included in the study and discharges were taken into account when calculating exposure times. Approval for the study protocol was obtained from the Ethics Committee of Pirkanmaa Hospital District on 11 April 2006.

### Physical training programme

At the beginning of military service, conscripts performed eight weeks of basic training consisting of varying physical activities, including marching, cycling, skiing, orienteering, swimming, drill training and combat training, or other training involving moderate or heavy physical loading. There was an average of 17 hours of military training per week with a gradual increase in intensity. During combat training and marching, conscripts usually carry approximately 26 kg to 36 kg of personal military equipment and, occasionally, an additional 5 kg to 20 kg of team military equipment. In addition, conscripts performed other physical exercises, such as jogging, team sports, and circuit training, for an average of seven hours per week. The basic training period was followed by diverse training programmes depending on the company and service duration. Over the following four months of service, however, the amount of moderate and high-intensity physical training was maintained approximately at the same level in the different companies.

### Musculoskeletal disorder registration

The data of the first arrival were collected from July 10^th ^2006 to January 5^th ^2007 and for the second arrival from January 8^th ^2007 to July 6^th ^2007. A musculoskeletal disorder (MSD) (including overuse and acute injuries) was defined as an event that resulted in physical damage to the body for which the conscript sought medical care from the garrison clinic. Heat or cold injuries were not included in the analysis. Only those wounds that were direct consequences of musculoskeletal contusions were considered MSDs. During military service, all conscripts had to use the services of the military healthcare units. The date, anatomical location, type, aetiological circumstances, severity and diagnosis of each MSD were registered in electronic patient records. Because the conscripts may have sought medical care several times due to the same MSD, the total number of health clinic visits exceeded the number of MSDs (Table 2). The health clinic visits were considered to be for the same disorder when the conscript had sustained an MSD of the same type and location during the preceding two weeks or if a physician had marked on the patient files that the reason for the visit was related to the previous MSD.

**Table 2:** Distribution of musculoskeletal disorders by anatomical location in 944 male conscripts during six-month military service.

Body part	Total number (%)	Acute/Overuse,%	Incidence* (95% CI)	Average number of health clinic visits per disorder
Lower extremity	1063 (65%)	26/74	6.9 (6.5-7.3)	1.8
Knee	315 (19%)	32/68	2.0 (1.8-2.3)	2.0
Ankle	192 (12%)	39/61	1.2 (1.1-1.4)	1.7
Foot	195 (12%)	8/92	1.3 (1.1-1.5)	1.9
Shin	103 (6%)	15/85	0.7 (0.5-0.8)	2.5
Back	300 (18%)	19/81	1.9 (1.7-2.2)	1.8
Low back pain	263 (16%)	18/82	1.7 (1.5-1.9)	1.8
Upper extremity	177 (11%)	56/44	1.1 (1.0-1.3)	1.5
Shoulder	87 (5%)	28/72	0.6 (0.5-0.7)	1.6
Head	32 (2%)	100/0	0.2 (0.1-0.3)	1.3
Other parts of body	57 (3%)	43/57	0.4 (0.3-0.5)	1.7
Total	1629 (100%)	30/70	10.5 (10.0-11.1)	1.8

Total number, proportions of acute and overuse-related disorders and their incidence and mean number of health clinic visits per disorder are given according to the anatomical location.

* Event-based incidence expressed as total number per 1000 person-days

The type of MSD was categorised as acute if the MSD had a sudden onset involving known trauma. Overuse-related MSDs had a gradual onset without known trauma [30,31]. For instance, overuse conditions of the knee, shin, ankle and foot were categorised as lower limb overuse injuries, whereas sprains, strains, wounds, internal knee ligament ruptures and joint dislocations were typically categorised as acute injuries.

Disorders that occurred during the conscript's leisure time or on the way to vacation or back to garrison were also included. After careful clinical examination, necessary diagnostic tests and radiological graphs, the most accurate diagnosis was selected by a physician according to the 10th Revision of the International Classification of Diseases and Related Health Problems (ICD-10). The type and anatomical location of the MSD was reported according to the diagnosis. The severity of the MSD was categorised according to the number of days of limited duty: 1-3 days denoting minimal, 4-7 days mild, 8-28 moderate MSD and more than 28 days severe MSD [31]. Limited duty involved a physical restriction that prevented the conscript from fully participating in military training events. Release from military service was indicated when a physician determined a conscript unable to continue military training. Releases from military service due to musculoskeletal injuries were registered as severe MSDs.

### Assessment of physical fitness

**Figure 2:**
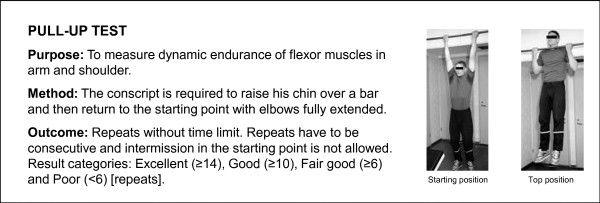
**Description of pull-up test**. The test is based on practice in the Finnish Defence Forces.

**Figure 3:**
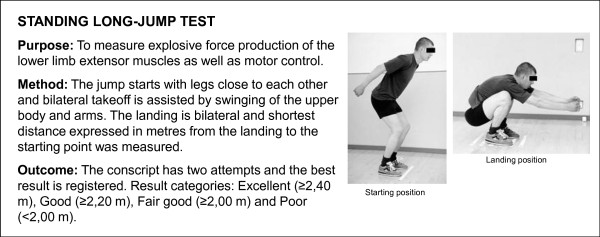
**Description of standing long jump test**. The test is based on practice in the Finnish Defence Forces.

**Figure 4:**
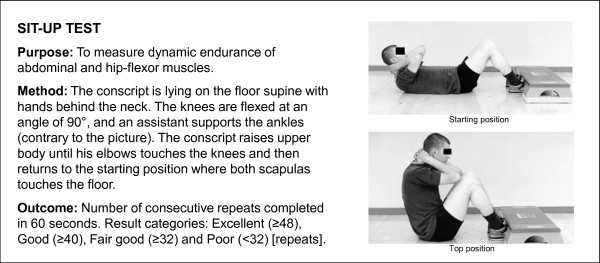
**Description of sit-up test**. The test is based on practice in the Finnish Defence Forces.

**Figure 5:**
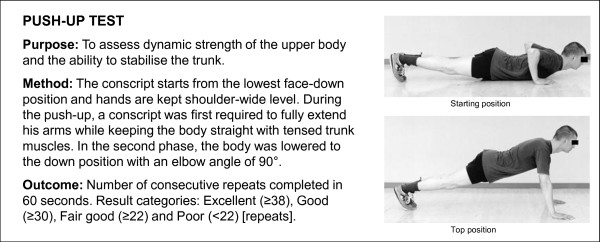
**Description of push-up test**. The test is based on practice in the Finnish Defence Forces.

**Figure 6:**
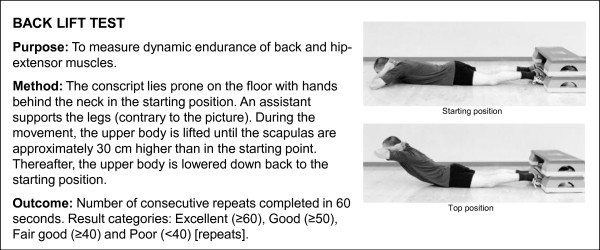
**Description of back lift test**. The test is based on practice in the Finnish Defence Forces.

A Cooper's test (12-minute running test) and muscular fitness tests were performed by most (98%) conscripts during their first weeks of military service. A minority of conscripts (2%) were unable to complete their physical fitness tests during the first two weeks due to minor health problems, such as infections or overuse injuries. Muscular fitness tests and the 12-minute run test were performed on different days. Muscular fitness tests included push-ups, sit-ups, pull-ups, the standing long jump and a back-lift test [32]. Instructors of the companies supervised so that each test was performed technically correctly. The recovery time between each muscle test was at least five minutes. For the pull-up, a conscript was required to raise his chin over a bar and then return to the starting point with elbows fully extended. For the standing long jump, a conscript started the jump with legs close to each other and bilateral take-off was assisted by swinging of the upper body and arms. The landing was bilateral and shortest distance expressed in metres from the landing to the starting point was measured. For the sit-up, a conscript was lying on the floor supine with hands behind the neck. The knees were flexed at an angle of 90°, and an assistant supported the ankles. The conscript raised the upper body until his elbows touched the knees and then returned to the starting position where both scapulas touched the floor. For the push-up, a conscript was first required to fully extend his arms while keeping the body straight with tensed trunk muscles. In the second phase, the body was lowered to the down position with an elbow angle of 90°. For the back lift, a conscript lay prone on the floor with hands behind the neck in the starting position and an assistant supported the legs. During the movement, the upper body was lifted until the scapulas were approximately 30 cm higher than in the starting point. Thereafter, the upper body was lowered down back to the starting position. More detailed information about physical fitness tests is presented in Figures 2, 3, 4, 5 and 6.

To calculate the muscle fitness index (MFI), the points from individual muscle fitness test results (push-ups, sit-ups, pull-ups, standing long jump, and back lift) were added together (Excellent = 13-15 points, Good = 9-12 points, Fair good = 5-8 points, Poor = 0-4 points). Poor result in individual muscle fitness test equated to zero points, a fair good result to one point, a good result to two points and an excellent result to three points. A conscript's physical fitness index (CPFI) was calculated using the following formula: (12 min running test result (metres) + 100 × MFI)/200 (Table 1, see footnotes 5 and 6). These formulas are based on standard practice in the Finnish Defence Forces since 1982 [33]. Because excellent results in Cooper's test were uncommon (<4%), the two highest levels, good and excellent, were combined to obtain a group of equal size for comparison. In addition, Cooper's and individual muscle fitness test results were combined into a single variable to explore whether the combined fitness variable, representing co-impairment, would be more strongly associated with the occurrence of MSDs.

**Figure 7:**
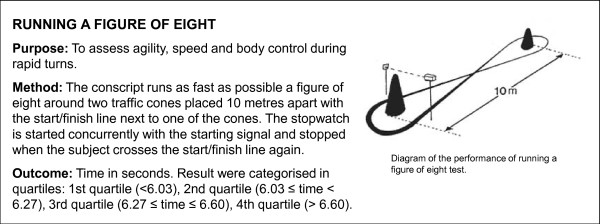
**Description of running a figure of eight test**. The test was performed for study purposes.

**Figure 8:**
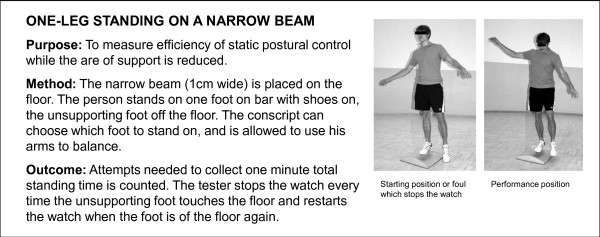
**Description of one-leg standing on a narrow beam test**. The test was performed for study purposes.

Two additional physical fitness tests of motor skill (running a figure of eight and standing on a narrow beam) were performed for study purposes (Figures 7 and 8). In addition, height, weight and waist circumference were measured during the first two weeks of service. Body mass index (BMI) was calculated by dividing weight (kilograms) with the square of height (metres). Waist circumference (WC) as a mark of abdominal obesity and excessive visceral fat [34] was measured with a tape at the midway between the lowest rib and iliac crest after normal exhalation. The cut-off points to describe overweight and obesity for BMI and WC were set according to the World Health Organization [35] (Table 3).

**Table 3:** Hazard ratios (HR) for musculoskeletal disorder (MSD) incidence and incidence of long-term MSD by health variables at baseline.

Health variable	Category	Total number (% of experienced MSD;% of experienced ≥10 service days lost due to MSDs)	HR for MSD incidence (n = 652) *	HR for MSD incidence (n = 652) **	HR for long-term MSD incidence (≥10 service days lost) (n = 194) *	HR for long-term MSD incidence (≥10 service days lost) (n = 194) **
Body mass index^1 ^(BMI = (kg)/(m)^2^)	Underweight (BMI < 18.5)	44 (66; 20)	1.1 (0.7-1.5)	1.1 (0.7-1.6)	1.1 (0.6-2.2)	1.1 (0.5-2.2)
	Normal (18.5 ≤ BMI < 25.0)	539 (67; 19)	1 (Referent)	1 (Referent)	1 (Referent)	1 (Referent)
	Pre-obese (25.0 ≤ BMI < 30.0)	220 (71; 19)	1.1 (0.9-1.3)	**1.2 (1.0**-**1.5)**	1.0 (0.7-1.5)	1.1 (0.7-1.6)
	Obese (BMI ≥ 30.0)	66 (82; 33)	**1.7 (1.3-2.3)**	**1.8 (1.3**-**2.4)**	**2.0 (1.3-3.2)**	**1.9 (1.2**-**3.2)**
						
Waist circumference (WC, cm)	Thin (WC < 80)	177 (64; 20)	1.0 (0.8-1.2)	1.0 (0.8-1.2)	1.2 (0.8-1.8)	1.1 (0.7-1.6)
	Normal (80 ≤ WC < 94)	499 (68; 17)	1 (Referent))	1 (Referent)	1 (Referent))	1 (Referent)
	Increased (94 ≤ WC < 102)	126 (74; 23)	1.2 (1.0-1.5)	1.2 (1.0-1.6)	1.4 (0.9-2.1)	1.3 (0.8-2.0)
	High (WC ≥ 102)	91 (79; 32)	**1.6 (1.2-2.0)**	**1.7 (1.3**-**2.2)**	**2.1 (1.4-3.3)**	**2.2 (1.3**-**3.5)**
						
Height (cm)	Shortest quartile (≤176)	184 (71; 24)	1 (Referent)	1 (Referent)	1 (Referent)	1 (Referent)
	Second quartile (177-180)	248 (63; 15)	0.8 (0.7-1.0)	0.9 (0.7-1.2)	**0.6 (0.4**-**0.9)**	0.7 (0.4-1.1)
	Third quartile (181-184)	212 (71; 20)	1.0 (0.8-1.3)	1.0 (0.8-1.3)	0.8 (0.6-1.3)	0.8 (0.5-1.2)
	Tallest quartile (≥184)	225 (72; 21)	1.0 (0.8-1.3)	1.1 (0.9-1.4)	0.9 (0.6-1.3)	0.8 (0.5-1.3)
						
Self-assessed health^2^	Good or very good	500 (66; 17)	1 (Referent)	1 (Referent)	1 (Referent)	1 (Referent)
	Average or inferior	434 (72; 24)	**1.3 (1.1-1.6)**	1.0 (0.9-1.3)	**1.6 (1.2-2.1)**	0.9 (0.7-1.3)
						
Sum factor of musculoskeletal symptoms	Minimal symptoms^3^	305 (62; 14)	1 (Referent)	1 (Referent)	1 (Referent)	1 (Referent)
	Mild symptoms^4^	357 (68; 21)	**1.2 (1.0-1.5)**	**1.4 (1.1**-**1.7)**	**1.7 (1.1-2.4)**	**1.9 (1.3**-**2.9)**
	Clear symptoms^5^	271 (78; 28)	**1.8 (1.5-2.2)**	**1.7 (1.3**-**2.1)**	**2.4 (1.7-3.6)**	**2.6 (1.7**-**3.9)**
						
Chronic disease	No	687 (68; 21)	1 (Referent)	1 (Referent)	1 (Referent)	1 (Referent)
	Yes	247 (72; 21)	1.2 (1.0-1.4)	1.1 (0.9-1.3)	1.0 (0.8-1.4)	1.1 (0.8-1.6)
						
Regular medication	No	834 (69; 21)	1 (Referent)	1 (Referent)	1 (Referent)	1 (Referent)
	Yes	96 (72; 18)	1.1 (0.9-1.4)	1.1 (0.8-1.4)	0.8 (0.5-1.4)	0.7 (0.4-1.3)
						
Orthopaedic surgery	Never	858 (68; 20)	1 (Referent)	1 (Referent)	1 (Referent)	1 (Referent)
	Yes	74 (73; 27)	1.2 (0.9-1.6)	1.1 (0.8-1.6)	1.3 (0.8-2.1)	1.4 (0.9-2.4)
						
Chronic impairment or disability^6^	No	789 (67; 19)	1 (Referent)	1 (Referent)	1 (Referent)	1 (Referent)
	Yes	140 (81; 31)	**1.6 (1.3-2.0)**	**1.4 (1.1**-**1.7)**	**1.8 (1.3-2.5)**	1.4 (0.9-2.1)
						
Sports injury during last month	No	842 (67; 20)	1 (Referent)	1 (Referent)	1 (Referent)	1 (Referent)
	Yes	88 (82; 25)	**1.4 (1.1-1.8)**	**1.4 (1.0**-**1.8)**	1.3 (0.8-2.0)	1.2 (0.7-2.0)

Variable distribution was charted in 944 male conscripts during the first week of military service and MSD outcomes were registered during the following six-month military service. Long-term MSD was defined as an incidence of time loss of at least 10 active service days due to one or several MSDs. Statistically significant findings are indicated with bold type.

* Adjusted for age (univariate).

** Adjusted for age, company, smoking, frequency of drunkenness before military service, baseline medical conditions (sports injury during the last month before military entry, chronic impairment or disability due to prior musculoskeletal injury, earlier musculoskeletal symptoms, chronic disease), school success (educational level and grades combined), father's occupation, opinion about physical demands for a soldier, urbanisation level of the place of residence, self-assessed health, waist circumference, height, participating in individual aerobic sports, last degree achieved in school sports, belonging to a sports club, self-assessed physical fitness, participation in competitive sports and physical activity during the previous three months before entering the military.

^1 ^Not adjusted by waist circumference since BMI and WC strongly interconnected (χ^2^-test, p < 0.001).

^2 ^Compared to age-mates.

^3 ^'Minimal symptoms': maximum seven-day lasting symptom in one anatomical region during the last month before entering the military.

^4 ^'Mild symptoms': symptoms in two to six anatomical regions, but the symptoms had lasted a maximum of one week during the last month before military entry.

^5 ^'Clear symptoms': included the remaining conscripts.

^6 ^Due to prior musculoskeletal injury.

### Pre-information questionnaire

A questionnaire was used to determine the conscripts' socio-economic factors (father's occupational group, school success and urbanisation level of the place of residence; Table 4), health (self-assessed health compared to age-mates, chronic disease, medication, previous orthopaedic surgeries and sport injuries, chronic impairment or disability and musculoskeletal pain in seven anatomical regions during the last month; Table 3) and health behaviour (use of alcohol and tobacco, frequency of drunkenness, opinion about physical demands of a soldier, amount of physical exercise, participation in individual aerobic sports, belonging to a sports club, participation in competitive sports, last degree achieved in school sports and self-assessed physical fitness; Table 5) at the baseline of the study just before entry to the military service. The questionnaires were performed during the first week of service.

**Table 4:** Hazard ratios (HR) for musculoskeletal disorder (MSD) incidence and incidence of long-term MSD by socio-economic variables and company at baseline.

Socioeconomic background & company	Category	Total number (% of experienced MSD;% of experienced ≥10 service days lost due to MSDs)	HR for MSD incidence (n = 652) *	HR for MSD incidence (n = 652) **	HR for long-term MSD incidence (≥10 service days lost) (n = 194) *	HR for long-term MSD incidence (≥10 service days lost) (n = 194) **
Father's occupational group	Not physical	325 (64; 20)	1 (Referent)	1 (Referent)	1 (Referent)	1 (Referent)
	Physical	416 (70; 20)	1.2 (1.0-1.4)	1.1 (0.9-1.4)	1.0 (0.7-1.4)	0.9 (0.6-1.3)
	Unclear or unemployed	185 (74; 24)	**1.3 (1.0-1.6)**	**1.3 (1.1**-**1.7)**	1.2 (0.8-1.7)	1.1 (0.7-1.7)
						
School success (combination of school type attended and school success)	Excellent^1^	138 (52; 12)	1 (Referent)	1 (Referent)	1 (Referent)	1 (Referent)
	Good^2^	410 (70; 18)	**1.7 (1.3-2.2)**	**1.4 (1.1**-**1.9)**	1.6 (0.9-2.7)	1.1 (0.6-1.9)
	Satisfactory^3^	319 (72; 24)	**1.9 (1.5-2.5)**	**1.5 (1.1**-**2.0)**	**2.3 (1.3**-**3.8)**	1.3 (0.7-2.4)
	Poor^4^	67 (81; 37)	**2.7 (1.9-3.9)**	**2.0 (1.3**-**3.0)**	**4.2 (2.2**-**7.7)**	**2.2 (1.1**-**4.5)**
						
Urbanisation level of the place of residence	≥10000 inhabitants	552 (70; 20)	1 (Referent)	1 (Referent)	1 (Referent)	1 (Referent)
	<10000 inhabitants	382 (66; 21)	1.0 (0.8-1.1)	0.9 (0.8-1.1)	1.1 (0.8-1.4)	1.0 (0.7-1.4)
						
Age	18-19 years	723 (68; 20)	1 (Referent)	1 (Referent)	1 (Referent)	1 (Referent)
	20-28 years	221 (71; 23)	1.1 (0.9-1.3)	1.1 (0.9-1.3)	1.2 (0.9-1.6)	1.2 (0.8-1.7)
						
Company	Anti-tank company	249 (61; 16)	1 (Referent)	1 (Referent)	1 (Referent)	1 (Referent)
	Signal company	234 (66; 16)	1.2 (1.0-1.5)	**1.3 (1.0**-**1.6)**	1.0 (0.7-1.6)	1.1 (0.7-1.8)
	Mortar company	69 (61; 9)	1.0 (0.7-1.4)	1.2 (0.8-1.7)	0.5 (0.2-1.2)	0.8 (0.3-1.9)
	Engineer company	215 (76; 24)	**1.5 (1.2-1.8)**	**1.5 (1.2**-**2.0)**	**1.6 (1.0-2.3)**	1.5 (0.9-2.4)
	Infantry company	100 (86; 36)	**2.1 (1.6-2.8)**	**1.9 (1.4**-**2.6)**	**2.6 (1.7-4.1)**	**2.6 (1.6**-**4.3)**
	Logistic company	61 (77; 34)	**1.7 (1.2-2.4)**	**1.7 (1.2**-**2.4)**	**2.4 (1.4-4.1)**	**2.2 (1.2**-**3.9)**
	Other companies^5^	16 (50; 0)	0.8 (0.4-1.7)	1.0 (0.5-2.1)	0.0 (0.0-∞)	0.0 (0.0-∞)

Variable distribution was charted in 944 male conscripts during the first week of military service and MSD outcomes were registered during the following six-month military service. Long-term MSD was defined as an incidence of time loss of at least 10 active service days due to one or several MSDs. Statistically significant findings are indicated with bold type.

* Adjusted for age (univariate).

** Adjusted for age, company, smoking, frequency of drunkenness before military service, baseline medical conditions (sports injury during the last month before military entry, chronic impairment or disability due to prior musculoskeletal injury, earlier musculoskeletal symptoms, chronic disease), school success (educational level and grades combined), father's occupation, opinion about physical demands for a soldier, urbanisation level of the place of residence, self-assessed health, waist circumference, height, participating in individual aerobic sports, last degree achieved in school sports, belonging to a sports club, self-assessed physical fitness, participation in competitive sports and physical activity during the previous three months before entering the military.

^1 ^Attended upper secondary school, polytechnic or university and reported excellent or good grades.

^2 ^Other subjects from upper secondary school, polytechnic or university and conscripts from vocational school whose grades were excellent or good.

^3 ^Respondents with poorer grades in vocational school.

^4 ^Attended only comprehensive school or had permanently interrupted vocational or upper elementary school.

^5 ^Conscripts were moved to different brigades.

**Table 5:** Hazard ratios (HR) for musculoskeletal disorder (MSD) incidence and incidence of long-term MSD by health behaviour variables at baseline.

Health behaviour	Category	Total number (% of experienced MSD;% of experienced ≥10 service days lost due to MSDs)	HR for MSD incidence (n = 652) *	HR for MSD incidence (n = 652) **	HR for long-term MSD incidence (≥10 service days lost) (n = 194) *	HR for long-term MSD incidence (≥10 service days lost) (n = 194) **
Smoking habits	Never smoked regularly	492 (62; 14)	1 (Referent)	1 (Referent)	1 (Referent)	1 (Referent)
	Has smoked regularly	439 (76; 28)	**1.5 (1.2-1.7)**	1.1 (0.9-1.3)	**2.1 (1.6-2.9)**	**1.5 (1.0**-**2.1)**
						
Use of alcohol	<1 time per month	176 (57; 14)	1 (Referent)	1 (Referent)	1 (Referent)	1 (Referent)
	1-2 times per week	603 (70; 21)	**1.3 (1.0-1.6)**	1.2 (0.9-1.5)	1.5 (1.0-2.2)	1.3 (0.8-2.1)
	≥3 times per week	154 (78; 25)	**1.7 (1.3-2.2)**	1.3 (1.0-1.9)	**1.8 (1.1-3.0)**	1.0 (0.5-1.9)
						
Frequency of drunkenness before military service	<1 time per week	723 (66; 19)	1 (Referent)	1 (Referent)	1 (Referent)	1 (Referent)
	≥1 time per week	211 (77; 27)	**1.4 (1.2-1.7)**	**1.3 (1.1**-**1.6)**	**1.6 (1.2-2.2)**	1.3 (0.9-1.8)
						
Agrees that soldier needs good physical fitness	Yes	598 (67; 19)	1 (Referent)	1 (Referent)	1 (Referent)	1 (Referent)
	No	336 (71; 23)	1.1 (1.0-1.3)	1.0 (0.8-1.2)	1.2 (0.9-1.6)	1.0 (0.7-1.3)
						
Sweating exercise (Brisk leisure time sport)	≥3 times per week	287 (62; 13)	1 (Referent)	1 (Referent)	1 (Referent)	1 (Referent)
	1-2 times per week	282 (72; 21)	**1.3 (1.1-1.6)**	1.2 (0.9-1.5)	**1.7 (1.1-2.5)**	1.2 (0.7-2.0)
	Only leisured exercise	183 (69; 24)	**1.4 (1.1-1.8)**	1.2 (0.9-1.6)	**2.1 (1.4-3.2)**	1.4 (0.8-2.3)
	No physical exercise	182 (75; 29)	**1.6 (1.3-2.0)**	1.2 (0.9-1.6)	**2.5 (1.7-3.9)**	1.3 (0.7-2.3)
						
Participates in individual aerobic sports	Yes, at least sometimes	638 (67; 18)	1 (Referent)	1 (Referent)	1 (Referent)	1 (Referent)
	No	293 (73; 26)	**1.2 (1.0-1.5)**	1.1 (0.9-1.3)	**1.6 (1.2-2.1)**	1.3 (0.9-1.8)
						
Belongs to a sports club	Yes, an active member	148 (64; 10)	1 (Referent)	1 (Referent)	1 (Referent)	1 (Referent)
	No	782 (70; 23)	**1.3 (1.0-1.6)**	**1.5 (1.1**-**2.0)**	**2.6 (1.5-4.4)**	**2.9 (1.4**-**5.8)**
						
Participates in competitive sports	Yes	138 (71; 16)	1 (Referent)	1 (Referent)	1 (Referent)	1 (Referent)
	No	794 (68; 21)	1.0 (0.8-1.2)	**0.7 (0.5**-**0.9)**	1.5 (0.9-2.3)	0.6 (0.3-1.1)
						
Last degree achieved in school sports	Very good or excellent	436 (67; 19)	1 (Referent)	1 (Referent)	1 (Referent)	1 (Referent)
	Good	301 (66; 20)	1.0 (0.9-1.2)	1.0 (0.8-1.2)	1.1 (0.8-1.5)	1.0 (0.7-1.4)
	Poor or fair	196 (76; 27)	**1.3 (1.1-1.6)**	1.0 (0.8-1.2)	**1.6 (1.1-2.3)**	0.8 (0.5-1.3)
						
Self-assessed physical fitness ^1^	Good or very good	217 (65; 14)	1 (Referent)	1 (Referent)	1 (Referent)	1 (Referent)
	Average or inferior	717 (70; 23)	**1.3 (1.1-1.6)**	1.0 (0.8-1.2)	**1.8 (1.2-2.6)**	1.1 (0.7-1.8)

Variable distribution was charted in 944 male conscripts during the first week of military service and MSD outcomes were registered during the following six-month military service. Long-term MSD was defined as an incidence of time loss of at least 10 active service days due to one or several MSDs. Statistically significant findings are indicated with bold type.

* Adjusted for age (univariate).

** Adjusted for age, company, smoking, frequency of drunkenness before military service, baseline medical conditions (sports injury during the last month before military entry, chronic impairment or disability due to prior musculoskeletal injury, earlier musculoskeletal symptoms, chronic disease), school success (educational level and grades combined), father's occupation, opinion about physical demands for a soldier, urbanisation level of the place of residence, self-assessed health, waist circumference, height, participating in individual aerobic sports, last degree achieved in school sports, belonging to a sports club, self-assessed physical fitness, participation in competitive sports and physical activity during the previous three months before entering the military.

^1 ^Compared to age-mates.

The school success variable was constructed as a combination of school type attended and grades achieved compared to an intermediate student in the class (Table 4), as follows: Excellent, attended an upper secondary school, polytechnic, or university and reported above average grades; Good, attended upper secondary school, polytechnic, or university and reported average or below average grades, or attended vocational schools and had above average grades; Satisfactory, attended vocational school and reported average or below average grades; Poor, attended only comprehensive school or had permanently interrupted vocational or upper elementary school.

Conscripts entering military service were young healthy men, all of whom had a medical check-up by a clinician during the 12 months before entering into the military. At the baseline, musculoskeletal symptoms during the last month before entry were assessed by a questionnaire. The sum factor of different musculoskeletal symptoms was developed by taking into account the questions about musculoskeletal pain and its severity in seven anatomical locations (neck, shoulder, forearm, low back, low back pain with radiation, hip, knee). Based on this factor, three different musculoskeletal symptoms categories were constructed (Table 3). Conscripts belonging to the 'minimal symptoms' category had symptoms lasting maximally for seven days in one anatomical region. The 'mild symptoms' category included conscripts who had pain in two to six anatomical regions, but the symptoms had not lasted longer than a week. The category of 'clear symptoms at least in one region' comprised the remaining conscripts.

### Statistical analysis

SPSS 17.0 for Windows software (SPSS Inc., Chicago, IL) was used for statistical analysis. MSD incidence was calculated by dividing the number of conscripts with one or more MSDs treated in the garrison clinic (numerator) for MSD by the total number of conscripts (denominator) and expressed as a percentage. Person-based incidence rate was calculated by dividing the number of conscripts treated in the garrison clinic for MSD by the exposure time. Exposure time for person-based incidence rate was calculated until onset of the conscript's first MSD. Event-based incidence rate was calculated by dividing the total number of MSDs by the exposure time. Exposure time for event-based incidence rate was calculated until the end of follow-up. Time loss due to MSD was allowed for when calculating the exposure time for the event-based incidence rate. The incidences with 95% confidence intervals (CI) were expressed per 1000 person-days. Descriptive statistics were used to analyse the data. To examine differences in the categorical baseline characteristics, the χ^2 ^statistics was used to test the hypothesis of no difference. Since continuous variables regarding baseline characteristics were not normally distributed, a Kruskall-Wallis test was used to test for a difference between the companies for continuous variables. A *P *value of < 0.05 was considered statistically significant.

Cox's proportional hazard models were applied to study the prospective associations between baseline characteristics and musculoskeletal disorder incidence (MSDI). The primary outcome was defined as an incidence of any type of MSD. The secondary outcome was defined as an incidence of time loss of at least 10 active service days due to one or several MSDs (hereafter referred to as a long-term MSDI). To examine the associations between risk factors and MSDs, continuous variables relating to physical fitness (Table 6) and body characteristics (Table 3) were converted into categorical variables. In the first phase of the Cox regression, each independent variable was analysed one at a time (univariate). Results were expressed as hazard ratios (HR) and calculated with 95% CIs with age at baseline forced into the model. A multivariate Cox regression was used to identify independent risk factors for MSDI and long-term MSDI and examine interactions between risk factors. Only possibly significant variables (*P *< 0.20) in the initial univariate-models were included in the multivariate model: company, father's occupational group, urbanisation level of the place of residence, self-assessed health, opinion about physical demands for a soldier, last degree achieved in school sports, belonging to a sports club and self-assessed physical fitness were included in the multivariate model as possible confounders. Smoking status (previous or current regular smoker), poor baseline medical condition (sports injury during the last month before military entry, chronic impairment or disability due to prior musculoskeletal injury, earlier musculoskeletal symptoms, chronic disease), not participating in individual aerobic sports and low physical activity during the previous three months before military entry were entered into the multivariate model as known risk factors. We considered poor school success (educational level and grades combined), participation in competitive sports, height and high frequency of drunkenness before military service as possible risk factors after univariate modelling and entered these variables into the multivariate model although the literature considering these variables as risk factors of MSDs during military training is sparse. In addition, high waist circumference and older age were considered possible risk factors and were therefore included in the multivariate model although results from previous studies are to some extent conflicting. A *P *value of < 0.05 was considered statistically significant when interpreting the results from Cox's proportional hazard models.

**Table 6:** Hazard ratios (HR) for musculoskeletal disorder (MSD) incidence and incidence of long-term MSD by physical fitness test variables at baseline.

Physical fitness test result	Category	Total number (% of experienced MSD;% of experienced ≥10 service days lost due to MSDs)	HR for MSD incidence (n = 652) *	HR for MSD incidence (n = 652) **	HR for long-term MSD incidence (≥10 service days lost) (n = 194) *	HR for long-term MSD incidence (≥10 service days lost) (n = 194) **
Running a figure of eight (three attempts, best time [seconds])	Fastest quartile (<6.03)	211 (64; 16)	1 (Referent)	1 (Referent)	1 (Referent)	1 (Referent)
	Mid 50% (6.03-6.60)	431 (69; 19)	1.2 (1.0-1.5)	1.3 (1.0-1.6)	1.3 (0.8-1.9)	1.2 (0.8-1.9)
	Slowest quartile (>6.60)	215 (71; 22)	**1.3 (1.0-1.6)**	1.2 (0.9-1.7)	1.4 (0.9-2.2)	1.2 (0.7-2.2)
						
One-leg standing on a narrow beam (attempts needed to one minute total standing time)	Best quartile (1)	201 (63; 17)	1 (Referent)	1 (Referent)	1 (Referent)	1 (Referent)
	Mid 50% (2-6)	439 (71; 18)	1.1 (0.9-1.4)	1.1 (0.9-1.3)	1.1 (0.7-1.6)	0.9 (0.6-1.4)
	Poorest quartile (≥7)	221 (69; 25)	1.2 (0.9-1.5)	1.0 (0.7-1.2)	**1.5 (1.0-2.3)**	1.1 (0.7--1.8)
						
Cooper's test (12-minute running test)	Excellent (≥3000 m) Good (≥2600 m)	36 (67; 13) 214 (62; 13)	1 (Referent)	1 (Referent)	1 (Referent)	1 (Referent)
	Fair good (≥2200 m)	435 (69; 20)	**1.2 (1.0-1.5)**	1.2 (0.9-1.5)	**1.5 (1.0-2.2)**	**1.6 (1.0**-**2.7)**
	Poor (<2200 m)	240 (76; 28)	**1.7 (1.4-2.1)**	**1.6 (1.2**-**2.2)**	**2.3 (1.5-3.5)**	**2.5 (1.4**-**4.5)**
						
Pull-up test (consecutive repeats without time limit)	Excellent (≥14)	107 (65; 14)	1 (Referent)	1 (Referent)	1 (Referent)	1 (Referent)
	Good (≥10)	140 (66; 16)	1.0 (0.7-1.4)	0.8 (0.5-1.1)	1.2 (0.6-2.2)	0.8 (0.4-1.8)
	Fair good (≥6)	266 (70; 18)	1.2 (0.9-1.5)	0.8 (0.6-1.2)	1.3 (0.7-2.3)	1.0 (0.5-1.9)
	Poor (<6)	421 (71; 25)	1.3 (1.0-1.7)	0.8 (0.6-1.2)	**2.0 (1.2-3.4)**	1.1 (0.6--2.2)
						
Standing long jump test (two attempts, best result)	Excellent (≥2,40 m)	141 (62; 13)	1 (Referent)	1 (Referent)	1 (Referent)	1 (Referent)
	Good (≥2,20 m)	251 (69; 20)	1.3 (1.0-1.7)	1.2 (0.9-1.6)	1.6 (0.9-2.7)	1.1 (0.6-1.9)
	Fair good (≥2,00 m)	311 (69; 20)	**1.3 (1.0-1.7)**	1.2 (0.9-1.6)	1.6 (1.0-2.7)	1.0 (0.6-1.8)
	Poor (<2,00 m)	231 (74; 26)	**1.6 (1.2-2.0)**	1.4 (1.0-1.9)	**2.3 (1.4-3.8)**	1.4 (0.7-2.6)
						
Sit-up test (repeats per 60 seconds)	Excellent (≥48)	122 (64; 16)	1 (Referent)	1 (Referent)	1 (Referent)	1 (Referent)
	Good (≥40)	221 (71; 17)	1.2 (0.9-1.6)	1.0 (0.8-1.4)	1.0 (0.6-1.8)	0.8 (0.4-1.5)
	Fair good (≥32)	328 (70; 22)	1.3 (1.0-1.7)	1.0 (0.7-1.3)	1.4 (0.9-2.3)	0.8 (0.5-1.5)
	Poor (<32)	263 (70; 24)	**1.4 (1.0-1.8)**	0.9 (0.7-1.3)	1.6 (1.0-2.6)	0.7 (0.4-1.4)
						
Push-up test (repeats per 60 seconds)	Excellent (≥38)	283 (70; 18)	1 (Referent)	1 (Referent)	1 (Referent)	1 (Referent)
	Good (≥30)	216 (64; 16)	1.0 (0.8-1.2)	0.8 (0.7-1.1)	0.9 (0.6-1.4)	0.7 (0.4-1.1)
	Fair good (≥22)	263 (68; 21)	1.0 (0.9-1.3)	0.8 (0.6-1.0)	1.2 (0.8-1.8)	0.7 (0.4-1.1)
	Poor (<22)	172 (76; 30)	**1.4 (1.1-1.8)**	1.0 (0.7-1.3)	**2.0 (1.4-3.0)**	1.0 (0.6-1.8)
						
Back lift test (repeats per 60 seconds)	Excellent (≥60)	450 (65; 18)	1 (Referent)	1 (Referent)	1 (Referent)	1 (Referent)
	Good (≥50)	195 (68; 20)	1.1 (0.9-1.4)	1.0 (0.8-1.3)	1.1 (0.8-1.6)	0.9 (0.6-1.4)
	Fair good (≥40)	197 (73; 20)	**1.2 (1.0-1.5)**	1.1 (0.9-1.4)	1.2 (0.8-1.7)	0.8 (0.5-1.3)
	Poor (<40)	92 (83; 32)	**1.8 (1.4-2.3)**	**1.5 (1.1**-**2.0)**	**2.0 (1.3-3.1)**	1.2 (0.7-2.0)
						
Conscript's muscle fitness index^1^	Excellent (13-15 points)	94 (61; 12)	1 (Referent)	1 (Referent)	1 (Referent)	1 (Referent)
	Good (9-12 points)	249 (66; 17)	1.3 (0.9-1.7)	1.2 (0.8-1.6)	1.5 (0.8-2.9)	1.2 (0.5-2.5)
	Fair good (5-8 points)	336 (72; 22)	**1.5 (1.1-2.0)**	1.2 (0.9-1.8)	**2.0 (1.1-3.8)**	1.2 (0.5-2.5)
	Poor (0-4 points)	255 (71; 25)	**1.6 (1.2-2.2)**	1.1 (0.8-1.7)	**2.6 (1.3-4.8)**	1.1 (0.5-2.7)
						
Conscript's physical fitness index^2^	Excellent (≥21,00)	37 (59; 8)	1 (Referent)	1 (Referent)	1 (Referent)	1 (Referent)
	Good (17.00-20.99)	270 (66; 16)	1.3 (0.8-2.0)	0.9 (0-6-1.4)	2.1 (0.6-6.6)	1.1 (0.3-3.7)
	Fair good (13.00-16.99)	420 (69; 21)	1.5 (1.0-2.4)	1.0 (0.6-1.6)	2.8 (0.9-9.0)	1.2 (0.3-4.1)
	Poor (<13.00)	196 (77; 28)	**2.0 (1.3-3.2)**	1.2 (0.7-2.0)	**4.4 (1.4-14.0)**	1.6 (0.4-5.8)
						
Combination of Cooper's and standing long jump test	Excellent^3^	77 (58; 9)	1 (Referent)	1 (Referent)	1 (Referent)	1 (Referent)
	Good^4^	335 (65; 19)	1.3 (0.9-1.8)	1.1 (0.8-1.6)	**2.2 (1.0-4.9)**	1.5 (0.6-3.3)
	Fair good^5^	394 (72; 20)	**1.6 (1.2-2.2)**	**1.5 (1.0**-**2.1)**	**2.5 (1.2-5.4)**	1.8 (0.8-4.1)
	Poor^6^	117 (79; 33)	**2.1 (1.5-3.0)**	**1.6 (1.0**-**2.6)**	**4.8 (2.2-10.8)**	**3.0 (1.2**-**7.8)**
						
Combination of Cooper's and push-up test	Excellent^3^	135 (64; 13)	1 (Referent)	1 (Referent)	1 (Referent)	1 (Referent)
	Good^4^	361 (67; 17)	1.2 (0.9-1.5)	1.1 (0.8-1.4)	1.3 (0.8-2.2)	1.3 (0.7-2.4)
	Fair good^5^	336 (70; 23)	**1.3 (1.0**-**1.7)**	1.0 (0.7-1.4)	**1.9 (1.1**-**3.1)**	1.4 (0.7-2.8)
	Poor^6^	91 (82; 36)	**2.3 (1.7**-**3.1)**	**1.8 (1.2**-**2.8)**	**3.6 (2.0**-**6.5)**	**2.8 (1.2-6.2)**
						
Combination of Cooper's and back lift test	Excellent^3^	171 (60; 12)	1 (Referent)	1 (Referent)	1 (Referent)	1 (Referent)
	Good^4^	437 (68; 20)	**1.3 (1.0**-**1.6)**	**1.3 (1.0**-**1.7)**	**1.8 (1.1**-**2.9)**	1.7 (1.0-3.0)
	Fair good^5^	272 (74; 22)	**1.5 (1.2**-**2.0)**	**1.4 (1.0**-**1.9)**	**2.0 (1.2**-**3.3)**	1.5 (0.8-2.8)
	Poor^6^	43 (91; 42)	**3.6 (2.5**-**5.2)**	**2.9 (1.9**-**4.6)**	**5.0 (2.6**-**9.3)**	**2.7 (1.2**-**5.9)**

Variable distribution was charted in 944 male conscripts during the first two weeks of military service and MSD outcomes were registered during the following six-month military service. Long-term MSD was defined as an incidence of time loss of at least 10 active service days due to one or several MSDs. Statistically significant findings are indicated with bold type.

* Adjusted for age (univariate).

** Adjusted for age, company, smoking, frequency of drunkenness before military service, baseline medical conditions (sports injury during the last month before military entry, chronic impairment or disability due to prior musculoskeletal injury, earlier musculoskeletal symptoms, chronic disease), school success (educational level and grades combined), father's occupation, opinion about physical demands for a soldier, urbanisation level of the place of residence, self-assessed health, waist circumference, height, participating in individual aerobic sports, last degree achieved in school sports, belonging to a sports club, self-assessed physical fitness, participation in competitive sports and physical activity during the previous three months before entering the military.

^1 ^Muscle fitness index (MFI) is the sum of individual muscle fitness test results including push-up, sit-up, pull-up, standing long jump and back muscle tests.

^2 ^Conscript's physical fitness index (CPFI) = (12 min running test result (m) + 100 × MFI)/200.

^3 ^Excellent or good result in Cooper's test and excellent result in standing long jump/push-up/back lift tests.

^4 ^Excellent result in standing long jump/push-up/back lift test and fair good or poor result in Cooper's test, or excellent result in Cooper's test and good, fair good, or poor result in standing long jump standing long jump/push-up/back lift test, or good result in Cooper's test and good or fair good result in standing long jump/push-up/back lift test, or fair good result in Cooper's test and good result in standing long jump test.

^5 ^Poorer results than aforementioned, except the combination of poor results in both tests.

^6 ^Poor result in Cooper's test and poor result in standing long jump/push-up/back lift tests.

## Results

### Incidence of musculoskeletal disorders

During the one-year study period (July 2006-July 2007), a total of 1629 MSDs and 2879 health clinic visits due to MSDs were registered in the garrison clinic. A total of 652 of 944 (69%) conscripts sustained one or more MSDs during the six-month service. Of these, 35% had one, 24% had two, 17% had three, 11% had four, 7% had five and 6% had from six to ten MSDs. A total of 194 (21%) conscripts suffered from long-term MSD (≥10 service days lost due to one or several MSDs). The event-based incidence rate for MSD was 10.5 (95% CI: 10.0-11.1) and the person-based incidence rate was 7.1 (95% CI: 6.6-7.7) per 1000 person-days, respectively. The MSD incidences for first (68%) and second (69%) arrival did not vary statistically significantly (*P *= 0.74).

### Type and anatomical location of musculoskeletal disorders

Most MSDs were in the lower extremities (65%) followed by the back (18%), upper extremities including shoulders (11%), head (2%) and other parts of the body (torso excluding back; 3%) (Table 2). The most common types of MSDs were lower limb overuse injuries (48%) and low back pain (16%). Overuse-related MSDs (70%) were more than twice as prevalent as traumatic MSDs (30%; Table 2).

### Severity, immediate causes and associated activities of musculoskeletal disorders

The majority (69%, n = 1119) of disorders were classified as minimal leading to a maximum three-day exemption from military training, while mild MSDs accounted for 20% (n = 328), moderate for 8% (n = 138) and severe for 3% (n = 44) of all cases. Fractures (n = 15), bone stress injuries (foot n = 7, shin n = 5, femur n = 2, calcaneus n = 1; total 15 cases), dislocations (n = 22) and internal knee injuries (n = 25) represented the most severe injuries and accounted for the majority of long-term exemptions from military training. Twenty-eight (3.0% of all) conscripts were released temporarily (for at least six months) from military service due to MSDs after the two-week run-in period.

MSDs occurred mostly (93%) during military training. Some (7%) occurred during vacations and four cases (0.3%) while travelling to vacation or back to the garrison. Of the immediate causes of acute MSDs, falling down (17%) and collision with an object (16%) were most commonly associated with MSDs. The following immediate causes were: tackling or struggling during sports exercise (5%), jumping (5%), malposition of foot during ground contact (4%), traffic accident (4%), slipping (4%) and being compressed between two objects (4%). In 12% of acute MSDs, the immediate cause remained unclear. Marching and running (36%) were the most common activities associated with overuse-related MSDs, followed by carrying and lifting loads (10%) and other organised physical exercise excluding marches and combat training (6%). For 27% of overuse-related MSDs, however, the associated activity remained unclear due to the gradual onset of the MSD.

### Risk factors of musculoskeletal disorders

Tables 3, 4, 5 and 6 show the distribution of variables and the hazard ratios of MSDI and long-term MSDI for various health (Table 3), socio-economic (Table 4), health behaviour (Table 5) and physical fitness variables (Table 6) in the univariate and adjusted models.

With regard to *health*, we observed a strong association between obesity and MSDs. A BMI over 30 increased the risk for MSDI (HR 1.8; 95% CI: 1.3-2.4) and long-term MSDI (HR 1.9; 95% CI: 1.2-3.2). In addition, the pre-obese category (25 ≤ BMI < 30) was associated with MSDI, but not with long-term MSDI. Abdominal obesity (WC over 102 cm) was associated with a 1.7-fold risk for MSDI (95% CI: 1.3-2.2) and a 2.2-fold risk for long-term MSDI (95% CI: 1.3-3.5). A low self-assessed health level compared to age-mates was associated with both outcomes in univariate models, but not after further adjustments. Of the baseline medical conditions, the sum factor of musculoskeletal symptoms was the strongest predictor for both outcomes with a dose-response relationship. In addition, chronic impairment or disability due to earlier musculoskeletal injury and earlier sport injuries were associated with MSDI (Table 3).

From the *socio-economic background *variables, a conscript's poor school success was associated with a two-fold risk for MSDI (95% CI: 1.3-3.0) and a 2.2-folded risk for long-term MSDI (95% CI: 1.1-4.5) (Table 4). In addition, father's occupation was associated with MSDI, but not with long-term MSDI. The company of the conscript was clearly associated with both outcome variables. During the 180 days of military service, the MSDI was lowest in the anti-tank and mortar companies and highest in the infantry company (Table 4).

With regard to *health behaviours*, there was a strong association between detrimental health behaviour factors and MSDs based on the univariate analysis, but after further adjustments these associations weakened (Table 5). Smoking, use of alcohol, frequency of drunkenness, physical inactivity, not participating in individual aerobic sports, not belonging to a sports club, low level of achievement in school sports and low self-assessed physical fitness were all associated with the both outcomes in univariate models. In the final model, however, only high frequency of drunkenness, not belonging to a sports club, and on other hand, participating in competitive sports were associated with MSDI. Present or former cigarette smoking and not belonging to a sports club were associated with the long-term MSDI in the final model (Table 5).

High hazard ratios of MSD were observed in those conscripts with low levels of *physical fitness test results *(Table 6). Each fitness test was associated with MSDI or long-term MSDI in univariate models (Table 6). However, after final adjustments, only the 12-minute running test (Cooper) maintained its significance for both MSDI (HR 1.6; 95% CI: 1.2-2.2) and long-term MSDI (HR 2.5; 95% CI: 1.4-4.5). In addition, the back lift test was associated with MSDI in the final model. Cooper's and individual muscle fitness test results were combined into one variable to explore whether co-impairment in aerobic and muscular fitness would increase the risk for MSDs. Combinations of poor fitness in Cooper's test and standing long jump, push-up and back lift tests proved to be the strongest predictors for both outcomes with a dose-response relationship. Poor results in both Cooper's and standing long jump test were associated with a 1.6-fold risk for MSDI (95% CI: 1.0-1.6) and 3.0-fold risk for long-term MSDI (95% CI: 1.2-7.8). Accordingly, poor results in both Cooper's and push-up test were clear predictors for both outcomes, HR being 1.8 (95% CI: 1.2-2.8) for MSDI and 2.8 (95% CI: 1.2-6.2) for long-term MSDI. In addition, poor results in both Cooper's and back lift test were strongly associated with MSDI (HR 2.9; 95% CI: 1.9-4.6) and long-term MSDI (HR 2.7; 95% CI: 1.2-5.9) (Table 6). Results of the pull-up or sit-up test combined with Cooper's test, however, were not significant for either outcome (data not shown).

## Discussion

In the present study, we examined risk factors for MSDs among male conscripts during a six-month military service. The findings indicated that a low level of physical fitness expressed by 12-minute running (Cooper's test) was clearly associated with MSD with a dose-response relationship, confirming the association of low levels of aerobic fitness and subsequent risk of injury [6-8,18,20-24,36,37]. Furthermore, we present new findings that poor results in standing long jump, push-up or back lift tests combined with poor result in Cooper's test are strong predictors for MSDs. In addition, higher WC and BMI, earlier musculoskeletal symptoms, poor school success and company were all clearly associated with MSDs elucidating previously equivocal findings. It was also observed that some military tasks specific to the company involve higher risks for MSDI than other tasks. Good entry-level physical fitness, normal BMI and normal WC were protective factors against MSDI in all companies suggesting that these intrinsic and modifiable risk factors are amenable for prevention programmes.

The main finding of the present study was the association between low physical fitness and MSDs. A number of studies have documented the association of low levels of aerobic fitness and subsequent risk of injury [6-8,18,20-24,36,37], although a conflicting result was reported in a Finnish study of injury hospitalisations [9]. Poor muscular strength and endurance are also reported to be risk factors for injuries during military training, although not as frequently [7,8,23,27]. A civilian study among intercollegiate basketball and track athletes clarified these findings by demonstrating that core stability has an important role in the prevention of lower extremity injuries [38]. The findings of the present study, that poor back lift or push-up test result combined with poor aerobic endurance (Cooper's test) are strong predictors for MSDs, support the importance of core strength and stability to protect against MSDs. Moreover, improved control of the lumbar neutral zone with trunk muscles decreases low back pain among middle-aged men [39], a common MSD in the present study.

The US Army Physical Fitness Test includes a two-mile (3.2 km) run and push-up and sit-up tests. Hence, the finding that MSDs were associated with poor results in standing long jump and back-lift tests is new. In the present study, a combination of Cooper's test and lower extremity muscle fitness (standing long jump test) proved to be a strong predictor for MSDs with a dose-response relationship. The standing long jump requires efficient motor control of the whole body in addition to measuring power production of the lower limb extensor muscles. Moreover, the standing long jump test is a good marker of lower limb dynamic muscle strength [40]. The present finding suggests that in addition to good aerobic endurance, motor control and strength of the lower extremities are important factors of physical fitness in the prevention of MSDs during military training. However, criticisms have been raised with regard to army physical fitness tests because they tend to penalise larger, not just fatter, individuals because body weight acts as a load. Larger individuals receive lower scores than their lighter counterparts, although larger persons perform work-related fitness tasks, such as carrying loads, better in a military environment [41].

Individuals with lower aerobic capacity probably experience greater physiological stress than individuals with better aerobic fitness during long-term military basic training (marching, running, combat training), which may also predispose to MSDs [1,7]. Various hypothetical mechanisms have been presented to explain this association. Conscripts with lower aerobic fitness levels may perceive military training as more difficult and fatigue more rapidly [42]. It has also been proposed that fatigue leads to changes in gait and kinematics in lower extremities [43,44] which may result in musculoskeletal stress in specific body areas and predispose to injuries [45].

Low levels of physical activity are associated with injuries in several military studies [3,7,11,21,37]. In the present study, low physical activity level during the three months prior to entering military service was associated with the risk of MSDI with a dose-response relationship, but only in the univariate models. This may be due to the fact that results in the final model were adjusted by other physical activity-related variables. Physical activity level before entry into the military service in particular, is associated with overuse injuries [10,20,23,36,46] suggesting that untrained conscripts overload their musculoskeletal structures and tissues more often than their active counterparts during military training.

Among young civilians, high exposure to competitive sports participation is associated with a higher risk of injuries [47,48], consistent with the findings of the present study. In previous military studies, however, participation in competitive sports was not associated with MSDs [6,21]. High running mileage is an evident risk factor for injuries based on several military [1,3,11,14-17] and civilian studies [49-51], indicating that as the total amount of exercise increases, the injuries decrease first, until a point is reached at which injuries increase disproportionately with changes in physical fitness [49].

In the present study, abdominal obesity and high BMI were associated with a higher risk for MSDI and long-term MSDI compared to smaller WC and normal BMI. In earlier studies, higher BMI was linked to an increased risk of injury during military service [6,9,26,46], although contradictory results indicating no association between BMI and injuries [24,50], and an association of lower BMI with injuries [21] are also reported. Mattila and colleagues [40] demonstrated that a high proportion of body fat measured by dual-energy x-ray absorptiometry (DEXA) is clearly associated with poor running performance and muscle strength among conscripts and proposed a stricter entry level BMI for Finnish conscripts. Morbidly obese persons might be temporarily discharged from the army in Finland, mainly on the basis of their subjective perception of being able to cope with military service [40]. Severely obese persons do not meet military entrance standards [7] in professional armies, which may partly explain the equivocal results from different studies.

Among the lifestyle characteristics, smoking, alcohol intake and frequency of drunkenness were clearly associated with MSDs in univariate models, but after further adjustments the associations weakened. The present finding that high frequency of drunkenness prior to the beginning of military service is a risk factor for MSDs has, to our knowledge, not been reported before. Risk taking behaviour and cognitive deficits are more common among smokers, which may partly explain the altered risk for MSDs in adjusted models [1,52]. Moreover, smoking and alcohol intake are strongly associated with each other among young men [53,54] which is consistent with the present data. This interaction attenuated the association between MSDs and predictive variables when both variables were placed in the same model. Altarac and colleagues [19] reported that cigarette smoking is associated with exercise-related injuries sustained during basic military training. After controlling for other factors, the adjusted odds ratio for smokers experiencing an exercise-related injury during basic military training was approximately 1.5-fold compared to non-smokers. Similar findings have also been reported in other military studies [3,11,18,25,28,37]. Although among young smokers, the aerobic capacity is similar to non-smokers [7], smoking may be associated with MSDs in many other ways. Smoking causes a deficit in bone density [55]. This effect may be detected even in young healthy persons [56]. Several studies have concluded that smoking hampers wound and fracture healing and impairs fibroblast function [57,58]. Overuse injuries are known to result from repetitive microtrauma leading to inflammation and local tissue damage [59]. There is no clear evidence, however, of the association between smoking and bone fractures among military recruits, because the underlying mechanisms are thought to depend on long-term exposure [19]. Overall, alcohol and smoking are probably indicators for risk-taking behaviour rather than causal risk factors for MSDs among the young during military training.

The finding of the present study that lower school success, a combination of educational level and grades in school, was associated with MSDs is concordant with some previous studies [12,60]. These studies reported lower educational level as a risk factor for foot injuries [12] and military discharge [60], but in general the association of poor school success and MSDs has not been investigated in the army setting. Lower grade of mental ability, however, is reported to be associated with acute musculoskeletal injuries [61] and severe low back pain [62] among young men.

It is well established that previous injury history is associated with a higher risk of injury during basic military training [3,11,14,46]. In the present study, chronic impairment or disability due to earlier musculoskeletal injury and prior sports injury during the month before military entry were also associated with a higher risk for MSD. On the other hand, a past training injury may be a marker of past physical activity [20]. Musculoskeletal symptoms during the three months before military entry were strongly associated with MSDs in the present study. This predictive association is not generally investigated in the army environment, but musculoskeletal complaints are associated with a higher risk for premature discharge from military service [28].

The results of civilian [63] and military [3,7,13,14,17] studies suggest that modification of running distance, frequency and duration may be effective toward preventing lower extremity injuries. A recent study by Finestone and Milgrom [17] reported a promising 60% decrease in stress fractures by reducing cumulative marching and by assuring a minimum sleep regimen in the Israeli army. Similar findings were reported in a previous study of soldiers in the US Army [4]. Both studies reported that these changes in military training did not lower the soldiers' combat readiness or physical fitness test results. The key element in military weight-bearing training to avoid overuse related MSDs is to gradually increase the distance, frequency and duration of training [3,13,14,23]. A study of the Singaporean army, however, demonstrated that a formal pre-training conditioning programme may be more effective toward reducing attrition than training with a gradual increase in pace, which extended the basic military training by one month [64]. Similar findings from the US Army showed that pre-conditioning of low-fit recruits resulted in lower attrition and a tendency towards lower injury risk [65]. In the Finnish Defence Forces, as well as in other mandatory armies in Nordic countries, the proportion of conscripts with low physical fitness and obesity has increased dramatically over recent decades. This phenomenon may cause serious health problems in the future. In addition, the phenomenon forces military training programmes to adapt to these changes in mandatory armies [32,40].

A recently published randomised controlled trial from the Danish conscription army revealed that an exercise programme enhancing muscular strength, coordination, and flexibility based on intrinsic risk factors identified in previous studies was not effective in reducing the incidence of lower extremity overuse injuries [66]. This study was the first randomised, placebo-controlled study investigating the preventive effect of concurrent exercise programmes on overuse injuries in the military environment. The intervention was speculated to be more effective in situations with a more gradual increase in load [66].

The present study has several strengths. First, the definition of MSD is clear. Moreover, the data regarding MSDs was collected using electronic patient files, which guaranteed a high coverage of MSDs because all patients who entered the garrison clinic were recorded in the computerised system. Second, the participation rate was high (98%). Furthermore, the design of the study was a prospective follow-up of two successive cohorts of conscripts with the aim of providing information on the risk factors of MSDs in an army environment during one entire year. The study limitations arise from the fact that, after the initial eight weeks of basic training, training programmes diverged depending on the company. Although the physical training was maintained at approximately the same level in different companies, the military training tasks were different. The presented associations between risk factors and MSDs were, however, adjusted by the company. In addition, because the threshold for seeking medical care may vary between individuals, some conscripts may have been more inclined to seek professional care than others.

The present study provides a wide spectrum of modifiable risk factors for MSDs. Although association does not indicate causality, increased knowledge of the risk factors and injury mechanisms is an essential component when planning intervention programmes. An appropriate intervention based on the results of the present study would be to increase both aerobic and muscular fitness prior to conscript training. Attention to appropriate waist circumference and BMI would strengthen the intervention programme. Well-planned randomised controlled studies are needed to provide more evidence from effective interventions before large-scale prevention programmes are initiated in a military environment.

## Conclusions

The findings of the present study provide a reliable insight into the intrinsic risk factors for MSDs. This study showed that a low cardiorespiratory fitness level expressed by poor results in a 12-minute running test at entry into the military service is strongly associated with MSD in a dose-response manner. Furthermore, we found that co-impairments in cardiorespiratory and muscular fitness (i.e., poor results in Cooper's test combined with a poor result in standing long jump, push-up or back lift tests) are the strongest predictors for MSDs. In addition, abdominal obesity, high BMI, earlier musculoskeletal symptoms, poor school success and physically demanding military training tasks are clearly associated with MSDs. The majority of the observed risk factors are modifiable and favourable for future interventions. The present results suggest that a good result (≥2600 m) in the 12-minute running test is a desirable goal in a pre-training programme before entering military service.

## Competing interests

The authors declare that they have no competing interests.

## Authors' contributions

HT participated in manuscript writing, data analysis, interpretation and data acquisition. JS was the primary investigator together with JP. They initiated and conceptually designed the study and participated in data processing and manuscript writing. HP participated in study concept and design as well as manuscript reviewing.

VMM took part in data analysis and interpretation and provided statistical expertise. He also participated in the study as a significant manuscript reviewer. OO took part in data analysis and interpretation. He also revised the manuscript critically and participated in the study concept and design. PV took part in designing the study and data acquisition. He also revised the manuscript critically. All authors have made substantive intellectual contributions to the study. All authors reviewed and approved the final manuscript.

## Pre-publication history

The pre-publication history for this paper can be accessed here:

http://www.biomedcentral.com/1471-2474/11/146/prepub
